# Can environmental regulations and R&D subsidies promote GTFP in pharmaceutical industry? Evidence from Chinese provincial panel data

**DOI:** 10.3389/fpubh.2022.1018968

**Published:** 2022-09-26

**Authors:** Yue-Di Yang

**Affiliations:** School of Economics, Shandong University of Finance and Economics, Jinan, China

**Keywords:** environmental regulations, government R&D subsidies, pharmaceutical industry, green total factor productivity, green development

## Abstract

Based on the panel data of 30 provinces in China's pharmaceutical industry from 2000 to 2019, this paper proposes to combine the super efficiency SBM model and GML productivity index to calculate the static and dynamic green total factor productivity (GTFP). Then, the Tobit model is adopted for regression analysis on how environmental regulations, government R&D subsidies, and their cross-terms affect the GTFP. Findings suggest that: (1) Static analysis reveals that the GTFP in China's pharmaceutical industry is markedly different among provinces and regions, and the dynamic analysis shows an upward trend from 2000 to 2019. (2) The GTFP of the pharmaceutical industry and environmental rules are connected in a U-shape. The government R&D subsidies to GTFP are positive and significant, and with the expansion of government R&D subsidies, the promotion effect of environmental regulations on GTFP is enhanced. Therefore, it is necessary to set up differentiated environmental regulations systems in different provinces and increase R&D subsidies to promote the pharmaceutical industry's green development.

## Introduction

**Figure 1:**
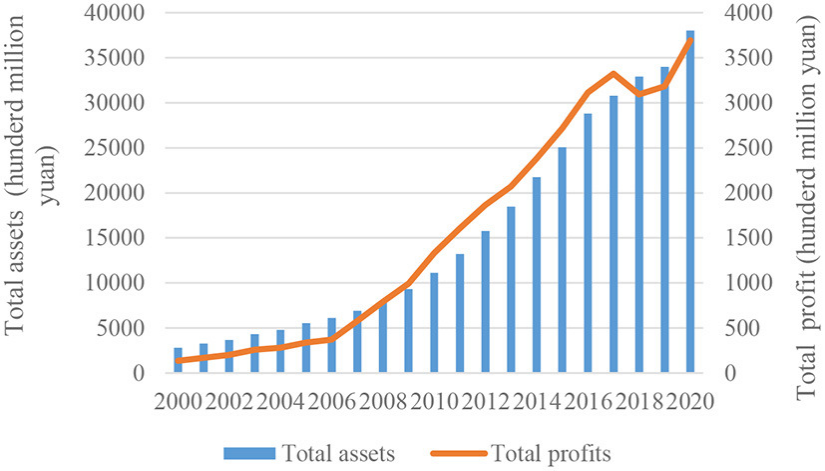
Total assets and total profits of the Chinese pharmaceutical industry.

The pharmaceutical industry is responsible for researching, developing, producing, and marketing pharmaceutical drugs, vaccines, and treatments for common and rare diseases. The demand for pharmaceutical products will gradually expand over the future years as the global population ages and health management knowledge rises (1). Hole et al. (2) point out that the pharmaceutical industry is one of the fastest-growing economic sectors with worldwide revenue. In recent years, due to increasing concerns on public health, China's pharmaceutical industry has made great progress, the scale of the industry has grown rapidly, the supply capacity has been significantly enhanced, and it occupies an important position in the national economy^1^. In 2010, China's pharmaceutical industry's production scale ranked third worldwide (3). Therefore, the market for pharmaceuticals in China is quickly becoming the second largest market in the world, only falling behind that of the United States in the year 2015 (4). The gap that currently exists in terms of the size of the pharmaceutical market between China and the United States will continue to get smaller over the course of the next few years (5). As shown in Figure 1, the total assets of the Chinese pharmaceutical industry have increased from 2,798.9 hundred million yuan in 2005 to 38,010.09 hundred million yuan in 2020, with the latter figure being ~13.58 times that of the former (6). Meanwhile, the total profits increased from 136.58 hundred million yuan in 2000 to 3,693.4 hundred million yuan in 2020. It can be seen that the development speed and quality of China's pharmaceutical industry has been significantly improved.

With the rapid development of the pharmaceutical industry, the problems related to resources and the environment has increasingly prominent. Melody (7) found that the carbon emissions of the pharmaceutical industry exceed those of the automotive industries. When it comes to mitigating the negative effects of detrimental impacts caused by energy consumption and environmental pollution, the pharmaceutical sector, which is one of the largest industries in the world, is a significant contributor to the problem. This is due to the fact that the pharmaceutical business requires inputs of energy, water, and raw materials, which results in significant volumes of consumption of energy and emissions of SO2 (8, 9). China's pharmaceutical industry has also posed a major threat to the natural environment in recent years. Historically, the energy consumption in China's pharmaceutical industry in 2,000 was only 9.77 million tons of standard coal equivalent, and this figure increased to 24.16 million tons of standard coal equivalent in 2019. Nearly 10,415 tons of SO2 were produced by the pharmaceutical industry in 2019. China has been striving to construct an energy-saving society. Facing the tremendous pressure of massive resource consumption and environmental pollution, China will pay more attention to reducing pollution emissions. Therefore, realizing energy-saving in the pharmaceutical industry is essential for China to achieve low-carbon transformation and pollution treatment (10, 11). At present, many scholars are implementing a new circular economy to reduce the adverse impact of pharmaceutical enterprises on the earth's ecosystem. That is, the pharmaceutical industry is leaning toward sustainable manufacturing (12). By enhancing the green total factor productivity (GTFP), we can encourage the high-quality and environmentally friendly growth of the Chinese pharmaceutical sector.

In order to alleviate environmental pressure, the Chinese government has issued a number of targeted policies and plans to encourage enterprises to carry out green innovation, improve GTFP and promote the development of green innovation (13). Environmental regulations are based on the prevention and treatment of environmental pollution. Through the conversion of production and consumption patterns, the negative externalities brought by economic activities to the environment will be minimized (14). Since 1979, when the Chinese government first promulgated the Environment in the People's Republic of China Protection Law (for trial implementation), a relatively complete system of environmental regulations has been established. Currently, this system includes ~26 relevant laws, more than 50 regulations, about 800 standards, and more than 660 normative legal documents, mainly covering pollution prevention and treatment and protecting natural resources. The recent environmental policy pointed out that China's pharmaceutical industry should adhere the green development and promote the green transformation upgrading of the pharmaceutical industry. However, due to the complex interaction of multiple factors, the relationship between environmental regulations and the pharmaceutical industry's development has not been determined. On the one hand, environmental regulations increase the investment and innovation cost of environmental pollution control in regulated industries, hindering the industry's short-term development (15). On the other hand, innovation compensation provided by environmental regulations will also encourage relevant enterprises to actively carry out various innovation activities (16). Many studies have shown that ecological investment and ecological innovation can be achieved through environmental regulations (17). It is of great significance to study the relationship between environmental regulations and GTFP in China's pharmaceutical industry. In the high-quality development of China's economy, whether environmental regulations can effectively reduce pollution emissions and promote the pharmaceutical industry's growth deserves penetrating analysis and discussion.

Furthermore, government R&D subsidies are also an important policy tool to affect the green innovation of enterprises (18). Acemoglu et al. (19) pointed out that the current market lacks enough incentives to encourage clean technology innovation, so enterprises must rely on government support. At present, there is no unified conclusion on the relationship between R&D subsidies and green enterprise innovation. Acemoglu et al. (20) believed that government subsidies could reduce the innovation risks of pharmaceutical enterprises and lay a solid foundation for technological innovation in this industry. Girma et al. (21) also found that government subsidies enhanced the competitiveness of the companies. But Lee et al. (22) argued that government subsidies increased entrepreneurial companies' initial investment cost because they spent the money on acquiring the subsidies. This means the negative effects of government subsidies also should not be neglected. The pharmaceutical industry has a long R&D period and is investment-intensive. It also has the characteristics of high-profit margins and high risks (23). As an industry with great economic contribution, high technology content, and closely related to people's livelihood, the Chinese government has highly valued the pharmaceutical industry. According to the financial data reported by the National Statistics Bureau of China, the amount of government R&D subsidies directed toward the pharmaceutical industry increased from 118.28 million yuan in 2000 to 2.92811 billion yuan in 2019. From the above analysis, this paper, based on the characteristics of the pharmaceutical industry, tries to explore the impact of government subsidies on GTFP in the pharmaceutical industry. Therefore, this paper attempts to calculate the static and dynamic GTFP of China's pharmaceutical industry and study whether environmental regulations and government R&D subsidies positively impact the GTFP of China's pharmaceutical industry.

The rest of the paper runs as follows. Section Literature review conducts a review of the literature. Section Methodology presents the research design. Section Variable and data description provides an overview of the data sources and indicator selection. Section Empirical results describes empirical and robustness tests, and analyzes our findings. The conclusions are presented in Section Conclusions.

## Literature review

### Environmental regulations and GTFP

One of the areas of study that academics have long concentrated on is the connection between environmental rules and the GTFP of the sector. Numerous academics have used a variety of techniques to examine how environmental rules affect GTFP in light of the worsening of environmental degradation. The Compliance Cost Hypothesis (24, 25) and the Porter Hypothesis are primarily the subject of the most recent studies on the effects of environmental regulations (26).

The Compliance Cost Hypothesis believed that the stricter environmental policies imply an extra burden for firms, including a shift of resources from the traditionally “productive” uses toward pollution abatement (27). The compliance costs are higher due to environmental regulations, and incentives for innovation are therefore weakened. As a result, productivity growth at the firm-level is likely to slow, at least in the short term (25, 28). Yuan and Xiang (29) used panel data from China's manufacturing industry and found that environmental regulations inhibited patent output and corporate innovation. Environmental rules, according to Zhao and Sun (30), hurt the competitiveness of businesses that produce a lot of pollution.

Porter (26) emphasized that although environmental regulations may increase the cost burden of enterprises, it provides enterprises with more opportunities for competitiveness. More importantly, reasonable and strict environmental regulations may lead to innovation and offset the cost of pollution treatment. This view is called Porter Hypothesis or Win-win Hypothesis. Initially, the research on Porter Hypothesis mostly focused on developed countries, which have experienced a long road of “grow first, cleaning up later” (31). Hamamoto (32) found that environmental commands and regulations increased innovations (R&D spending). Thus, boosting the productivity growth of five manufacturing samples in Japan over the past 20 years. In 18 OECD nations, De Santis et al. (33) discovered that various environmental restrictions have distinct but advantageous effects on labor and productivity growth. Many Chinese academics today have researched the Porter Hypothesis in greater depth. The Porter Hypothesis was confirmed by Zhang et al. (34), who found that China's carbon emission pricing system increased the effectiveness of green development in pilot provinces. The majority of studies revealed that various environmental restrictions had an impact on GTFP. The relationships between these effects could be linear (35), U-shaped (36), or inverted U-shaped (37). Based on the panel data of China's industrial sector, Ouyang et al. (38) found a U-shaped relationship between environmental regulation and technological innovation. Wang et al., (39) adopted the dynamic GMM model to confirm that an inverted U-shaped relationship between environmental regulations and green productivity.

### Government R&D subsidies and GTFP

The Chinese government has invested heavily in R&D subsidies to promote green innovation, the adoption of cutting-edge green technology, the acquisition of environmentally friendly machinery, the acceleration of mergers and acquisitions, and the improvement of the country's inefficient industrial structure on the way to its desired end result of green and sustainable development (40). Due to the important role of subsidies in government intervention, their impact on efficiency has attracted extensive attention from scholars. However, the results from previous literature are quite controversial due to the differences in countries, regions, sectors, and firms. The relationship between government R&D subsidies and efficiency are several controversial ideas (41).

Most academics agree that government R&D subsidies successfully address externalities in innovation (42, 43) and can improve GTFP (44). Specifically, because the creation of new technologies frequently necessitates a significant amount of funds, government R&D subsidies can compensate and encourage enterprises who engage in green innovation. Jourdan and Kivleniece (45) believed public R&D subsidies could help firms buffer their resource constraints during innovation. Wang et al. (46) pointed out that green insurance subsidies and government subsidies compensate for firms' funds shortage in green innovation, increasing enterprises' innovation willingness. Liu (47) also supported that government subsidy, an important source of funds, helps companies overcome capital shortage constraints. In addition, government R&D subsidies can mitigate information asymmetries and reduce the risk caused by R&D failure. Chapman and Hewitt-Dundas (44) argued that R&D subsidies could also improve enterprises' risk tolerance, enhancing the deepening of R&D activities. Research and development (R&D) subsidies, in the opinion of Bi et al. (48), have the potential to direct the direction of green R&D and reduce risks for companies who need to implement environmentally friendly innovations right away in order to comply with environmental regulations.

Despite the good side, many studies have shown that government R&D subsidies may have a negative impact on GTFP. This is because after receiving subsidies, enterprises may increase their dependence on subsidies and lack the motivation to work efficiently. Nilsson (49) found that Rent-seeking behavior may cause firms to reallocate resources to the process of seeking support, thus hindering efficiency improvement. Moreover, Dimos and Pugh (50) hold that the government R&D subsidies may completely or partially crowd out private R&D investment, which will restrict patent outputs and the innovation performance of enterprises. Varela-Candamio et al. (51) examined the effect of public subsidies on farming efficiency in Spain in 2013. The result demonstrated that public subsidies have a negative impact on the technical efficiency of the Spanish agricultural sector. This is because farmers are highly dependent on subsidies as a source of income, and government subsidies lead to low efficiency of farmers.

## Methodology

### Super efficiency SBM model with undesirable outputs

Studying GTFP in two dimensions (input and output) at the same time is made possible by the non-radial DEA model of the super efficiency SBM proposed by Tone (52).

Recommended the use of this concept. Super efficiency SBM model, in contrast to radial DEA, incorporates the slack factors. To account for the restrictions of radial measurement and to distinguish between numerous efficient decision-making units, the super-efficiency SBM model can be applied (DMUs). Almost always, unintended byproducts are developed during the process of utilizing energy, which leads in the production of polluting emissions. It wasn't until Tone (52) introduced his unsatisfactory super efficiency SBM model that undesired results were seriously considered in SBM research. This allows the model to more accurately depict the core concept underlying efficiency assessment. The model is described in greater detail below: Assuming there are n DMUs, with m inputs apiece, we can write the desired outputs as s_1_, and the unintended ones as s_2_. The input-output matrix contains $X=\left[x_{1}\cdots x_{n}\right]\in R^{m\times n},Y^{d}=\left[y_{1}^{d}\cdots y_{n}^{d}\right]\in R^{s_{1}\times n},Y^{u}=\left[y_{1}^{u}\cdots y_{n}^{u}\right]\in R^{s_{2}\times n}$. The super efficiency SBM model's expression with undesirable outputs is provided below.


(1) $$\rho^{*}=\frac{\frac{1}{m}\;\sum_{i=1}^{m}\left(\frac{\bar{x}}{xik}\right)}{\frac{1}{\left(s_{1}+s_{2}\right)}(\sum_{r=1}^{s_{1}}\frac{\bar{y^{d}}}{y_{rk}^{d}}+\;\sum_{t=1}^{s_{2}}\frac{\bar{y^{u}}}{y_{rk}^{u}}\;)}$$

$$s.t.\{\begin{array}{l} \bar{x}\geq \sum_{j=1,\neq k}^{n}x_{ij}\lambda_{j};i=1,2,\cdots m\\ \bar{y^{d}}\leq \sum_{j=1,\neq k}^{n}y_{rj}^{d}\lambda_{j};r=1,\cdots ,s_{1}\\ \bar{y^{u}}\leq \sum_{j=1,\neq k}^{n}y_{tj}^{u}\lambda_{j};t=1,\cdots ,s_{2}\\ \lambda_{j}\geq 0,j=1,2,\cdots n,j\neq 0\\ \bar{x}\geq xik;\bar{y^{d}}\leq y_{rk}^{d};\bar{y^{u}}\geq y_{tk}^{u} \end{array}$$

In the formula the slack variables of input are: $\bar{x}$,$\bar{y^{d}}$ and $\bar{y^{u}}$, desirable output and undesirable output, respectively; the weight vector is λ_*j*_; and ρ^*^ is the optimal solution of the model whenρ^*^≥1, the DMU is effective.

### Global malmquist-luenberger index model

The DEA approach, upon which the Malmquist-Luenberger index methodology is based, was designed to measure the degree to which the productivity of a single decision unit shifted over the course of varying time periods. The Global Malmquist-Luenberger (GML) index is built from the ground up using the direction distance function (DDF) as its foundation. This article provides the following definition of the output-directed DDF:


(2) $$\begin{array}{l} {\vec{D}}_{0}\left(x,y^{g},y^{b};g_{{}^{y}},g_{b}\right)=max\left\{\beta |\left(y^{g}+\beta g_{y},y^{b}-\beta g_{b}\right)\in P\left(x\right)\right\} \end{array}$$

Where *g* = (*g*_*y*_, *g*_*b*_) direction vector indicates that the expected output increases as much as possible in the gy direction and minimizes undesired output in the *g*_*b*_ direction. β is the highest possible ratio that can be attained by simultaneously raising the desirable output and lowering the undesirable output.

The ML productivity index can be produced using DDF based on output, according to Chung et al. (53). It is further subdivided into efficiency change index (EC) and technological change index (TC). Based on *t* period, *t*+1 period, the ML index is as follows:


(3) $$\begin{array}{l} ML_{t}^{t+1}=\left\{\frac{\left[1+{\vec{D}}_{0}^{t}(x^{t},y^{gt},y^{bt};g^{t})\right]}{\left[1+{\vec{D}}_{0}^{t}(x^{t+1},y^{g(t+1)},y^{b(t+1)};g^{t+1})\right]}\times \frac{\left[1+{\vec{D}}_{0}^{t+1}(x^{t},y^{gt},y^{bt};g^{t})\right]}{\left[1+{\vec{D}}_{0}^{t+1}(x^{t+1},y^{g(t+1)},y^{b(t+1)};g^{t+1})\right]}\right\}\\ \;=\frac{\left[1+{\vec{D}}_{0}^{t}(x^{t},y^{gt},y^{bt};g^{t})\right]}{\left[1+{\vec{D}}_{0}^{t+1}(x^{t+1},y^{g(t+1)},y^{b(t+1)};g^{t+1})\right]}\times \frac{\left[1+{\vec{D}}_{0}^{t+1}(x^{t},y^{gt},y^{bt};g^{t})\right]}{\left[1+{\vec{D}}_{0}^{t}(x^{t},y^{gt},y^{bt};g^{t})\right]}\times \frac{\left[1+{\vec{D}}_{0}^{t+1}(x^{t+1},y^{g(t+1)},y^{b(t+1)};g^{t+1})\right]}{\left[1+{\vec{D}}_{0}^{t}(x^{t+1},y^{g(t+1)},y^{b(t+1)};g^{t+1})\right]}\\ \;=EC_{t}^{t+1}\times TC_{t}^{t+1} \end{array}$$

The production frontier under the restrictions of safety regulation, as well as the shift in maximum actual output from period to period, are where efficiency change is reflected (catch-up). $ML_{t}^{t+1}$, $EC_{t}^{t+1}$ and $TC_{t}^{t+1}$ being greater than (less than) 1 signifies growth (decline) in total factor productivity as well as improvement in efficiency (deteriorations) and technical advancement (regression).

Oh (54) used *P*^*G*^(*x*) the global production possibility set and the global direction distance function to construct the GML index. GML index is based on the *P*^*G*^(*x*), can successfully avoid linear programming's flaw of having no solution. The prospect of the production front moving inward is eliminated by this continuous production frontier, which also avoids the occurrence of the technical reversal phenomena and, as a result, the passive growth of total factor productivity. This is a very important advantage. The following succinct definition of the global direction distance function:


(4) $$\begin{array}{l} {\vec{D}}^{G}\left(x,y^{g},y^{b};g_{{}^{y}},g_{b}\right)=max\left\{\beta |\left(y^{g}+\beta g_{y},y^{b}-\beta g_{b}\right)\in P^{G}\left(x\right)\right\} \end{array}$$

The GML index is defined as follows:


(5) $$\begin{array}{l} GML_{t}^{t+1}=\frac{\left[1+{\vec{D}}^{G}(x^{t},y^{gt},y^{bt};g^{t})\right]}{\left[1+{\vec{D}}^{G}(x^{t+1},y^{g(t+1)},y^{b(t+1)};g^{t+1})\right]}\\ \;=\frac{\left[1+{\vec{D}}^{t}(x^{t},y^{gt},y^{bt};g^{t})\right]}{\left[1+{\vec{D}}^{t\text{+}1}(x^{t+1},y^{g(t+1)},y^{b(t+1)};g^{t+1})\right]}\times \frac{\left[1+{\vec{D}}^{G}(x^{t},y^{gt},y^{bt};g^{t})\right]/\left[1+{\vec{D}}^{t}(x^{t},y^{gt},y^{bt};g^{t})\right]\;}{\left[1+{\vec{D}}^{G}(x^{t+1},y^{g(t+1)},y^{b(t+1)};g^{t+1})\right]/\left[1+{\vec{D}}^{t+1}(x^{t+1},y^{g(t+1)},y^{b(t+1)};g^{t+1})\right]\;}\\ \;=EC_{t}^{t+1}\times TC_{t}^{t+1} \end{array}$$

### Tobit model

Tobin (55) came up with the Tobit model, which limits the explained parameter in the range between 0 and 1 and conducts estimation by the maximum likelihood method. The Tobit model ensures the regression accuracy when the explained parameter is censored. By establishing the Tobit model to explore the impact of environmental regulations and government R&D subsidies on the GTFP. This study selects the GTFP as the explained variable, takes environmental regulations, government R&D subsidies, and their cross-terms as the core explanatory variables selects some control variables to establish three Tobit regression models.

The existing research shows that the relationship between the environmental regulations and GTFP is not certain but presents non-linear characteristics. The environmental regulations quadratic component is added to the analytical model since this paper contends that there is a U-shaped link between GTFP and environmental regulation intensity. The model looks like this:


(6) $$\begin{array}{l} GTFPi,t=\beta 0+\beta 1eri,t+\beta 1er_{i,t}^{2}+\beta 2controli,t+\varepsilon i,t \end{array}$$

Government R&D subsidies can make up for the lack of R&D investment and affect GTFP. Therefore, government R&D subsidies are added to Model (6) to construct a model (7). The model is as follows:


(7) $$\begin{array}{l} GTFP_{i,t}=\beta_{0}+\beta_{1}er_{i,t}+\beta_{1}er_{i,t}^{2}+\beta_{2}grd_{i,t}+\beta_{3}control_{i,t}+\varepsilon_{i,t} \end{array}$$

To study the impact of the simultaneous implementation of environmental regulations and government subsidies on the GTFP in China's pharmaceutical industry, add the interactionitem of environmental regulations and government R&D subsidies into the model (7), and build a model (8). The model is as follows:


(8) $$\begin{array}{l} GTFPi,t=\beta 0+\beta 1eri,t+\beta 1er_{i,t}^{2}+\beta 2grdi,t+\beta 3eri,t\\ \;*grdi,t+\beta 2controli,t+\varepsilon i,t \end{array}$$

Among them *GTFPi, t* represents the static GTFP, where its specific value has been calculated by the Super efficiency SBM model. *eri, t*represents environmental regulations. *er*_*i, t*_ represents the government R&D subsidies. *controli, t* is the control variable and ε*i, t* is a perturbation term.

## Variable and data description

### Variable description

#### Explained variable: GTFP

The GTFP seeks to strike a balance between the socioeconomic and ecological and environmental benefits of its initiatives. It is necessary for it to take into consideration how well-production processes use the various input components. At the same time, consideration is given to the resource environment, even though this comes at the expense of the product that is not desired.

Based on the existing literature on the selection of GTFP indexes, the indices selected in this study include four input indices, two desirable outputs, and one undesirable output. The inputs indicators include labor, capital, and energy. Because the pharmaceutical industry is extremely reliant on knowledge and technology. Therefore, a high-level R&D team is critical for the implementation of GTFP. Labor is measured by the indicator of technical R&D personnel in the pharmaceutical industry (56). The input of capital investment resources usually refers to the internal R&D expenses and new product development expenses (57). The total consumption of all energy sources, including coal, coke, crude oil, kerosene, gasoline, diesel, natural gas, fuel oil, and electricity, is used to compute energy consumption. Knowledge and economic output are examples of desirable outcomes, however there are also unintended consequences that can result from any given output. This study takes the number of patent applications revenue from new product sales and new product development expenses as desirable Output indicator variables. Discharge the amount of SO2 emissions as undesirable output indicators. In conclusion, this paper builds an indicator system for inputs and outputs required for GTFP; please refer to Table 1 for further details.

**Table 1:** Input and output indexes.

**Indicator index**	**Classification**	**Index composition**
Inputs indicators	Labor input	Employment in various regions
	Capital investment input	Internal R&D expenses
		New product development expenses
	Energy input	Total energy consumption
Outputs indicators	Outputs indicators	Number of patent applications
		Revenue from new product sales
	Undesirable Outputs indicators	SO_2_ emissions

The GML productivity index measures the growth rate and is a Chain index, reflecting the change degree of GTFP compared with the previous year. But the panel model analysis needs the year-on-year index, the actual GTFP. Therefore, this paper selects the GTFP measured by the super efficiency SBM model as the explanatory variable.

#### Core explanatory variables: Environmental regulations and government R&D subsidies

There is no set tool for regulating the environment, which makes it hard to measure. Some researchers measure environmental regulation with the investment ratio in industrial pollution control and total industrial output value. This paper studies the effect of environmental regulation from a regional perspective, so the ratio of investment in environmental pollution control and the GDP of each province is used to measure environmental regulation, which can reflect the overall level of environmental regulation from a macro perspective (58). Following the past practice of most scholars, this study takes the government R&D expenditures is chosen as the grd proxy variable.

#### Environmental rules and government subsidies are likewise distinct between areas and businesses because of these regional and business-specific variances

This study presents four control variables as a means of reducing the significance of the aforementioned disparities. The first is Openness to the outside world (Z1). Saggi (59) foreign enterprises, it was said, contributed capital and technology to the host country, making them more efficient than domestic firms. We introduce it as a control variable because varying degrees of openness to the outside world cause variances in the level of development, which in turn causes discrepancies in our estimates. It is calculated as the ratio of the total number of goods imported by each province to each region's gross domestic product (10,000 dollars, translated according to the annual average exchange rate of China's yuan against the US dollar) (10,000 yuan). Urbanization (Z2) is the second component. What we mean by “urbanization” reflects both the trend toward and the extent of people living in urban areas. This process will have far-reaching effects on both the economy and the environment. This article uses the number of permanent residents in each province and major city as a proxy for urbanization. Economic development level (Z3) is the third variable, Because China is such a large country, its regions vary greatly in terms of their levels of economic development. The corresponding environmental regulatory and government R&D subsidies effects also vary. The GDP per capita of each province serves as our yardstick for gauging economic development. The fourth variable, enterprise size (Z4), has a significant impact on GTFP since various enterprises of different sizes have varied motives for innovation. This variable's expression is the logarithm of the ratio of the number of industrial firms in each region over the required size to their main business income.

### Data sources

This analysis chooses provincial-level regional panel data for 30 Chinese provinces and cities from 2000 to 2019 based on availability (excluding Tibet, Hong Kong, Macao, and Taiwan, because of a lack of data). The data come from *China Statistical Yearbook, China Environmental Statistics Yearbook, China Science and Technology Statistics Yearbook*, and *China Energy Statistics Yearbook*. The few missing data of some parameters were completed through the moving average method.

For the purpose of decreasing the effect that multicollinearity and outliers exert on the stability of the model, the principal continuous variables were logarithmically processed. The explanatory variables are tested for multicollinearity; no multicollinearity exists between any two variables.

## Empirical results

### Static analysis of GTFP in China's pharmaceutical industry

This paper adopted the super-efficiency SBM model, which includes undesirable outputs, and calculates the GTFP of the pharmaceutical industry in provinces and regions^2^ based on the selected input and output indicators from 2000 to 2019. To balance China's socio-economic development, China divided its provinces into east, central, west, and northeast regions to implement different policies. The MaxDEA software is used to determine input and output indicators with the model, and the results are shown in Table 2.

**Table 2:** GTFP of China's provincial pharmaceutical industry from 2000 to 2019.

**Id**	**2000**	**2005**	**2010**	**2015**	**2016**	**2017**	**2018**	**2019**	**Mean**
Beijing	0.703	0.778	0.860	0.932	0.939	0.970	0.988	1.004	0.874
Tianjin	0.631	0.798	0.918	0.943	0.950	0.945	0.927	0.943	0.889
Hebei	0.684	0.630	0.655	0.749	0.746	0.769	0.778	0.807	0.687
Shanghai	0.783	0.759	0.809	0.885	0.892	0.907	0.927	0.923	0.830
Jiangsu	0.845	0.874	0.883	0.924	0.959	0.975	0.987	1.095	0.892
Zhejiang	0.750	0.740	0.826	0.920	0.930	0.932	0.934	0.944	0.841
Fujian	0.624	0.804	0.837	0.872	0.914	0.926	0.930	0.968	0.811
Shandong	0.630	0.699	0.770	0.888	0.903	0.900	0.891	0.910	0.782
Guangdong	0.753	0.773	0.810	0.919	0.929	0.920	0.936	0.929	0.828
Hainan	0.634	0.485	0.658	0.847	0.751	0.860	0.835	0.749	0.690
Shanxi	0.512	0.703	0.583	0.706	0.680	0.700	0.724	0.728	0.666
Anhui	0.583	0.672	0.734	0.846	0.887	0.940	0.977	0.962	0.757
Jiangxi	0.67	0.727	0.71	0.777	0.77	0.764	0.762	0.768	0.717
Henan	0.644	0.661	0.723	0.709	0.735	0.772	0.804	0.819	0.703
Hubei	0.615	0.612	0.806	0.895	0.892	0.891	0.894	0.930	0.752
Hunan	0.637	0.650	0.702	0.893	0.927	0.909	0.899	0.886	0.767
Inner Mongolia	0.450	0.660	0.322	0.579	0.659	0.660	0.656	0.655	0.582
Guangxi	0.711	0.707	0.727	0.788	0.796	0.797	0.806	0.822	0.747
Chongqing	0.713	0.887	0.818	0.940	0.956	0.956	1.000	0.982	0.873
Sichuan	0.596	0.673	0.740	0.801	0.776	0.803	0.845	0.843	0.724
Guizhou	0.676	0.632	0.724	0.755	0.787	0.815	0.850	0.786	0.747
Yunnan	0.654	0.689	0.706	0.714	0.717	0.747	0.767	0.724	0.709
Shaanxi	0.491	0.673	0.689	0.697	0.747	0.739	0.736	0.836	0.719
Gansu	0.577	0.625	0.594	0.671	0.674	0.710	0.735	0.629	0.651
Qinghai	0.208	0.290	0.415	0.489	0.468	0.531	0.475	0.554	0.402
Ningxia	0.129	0.207	0.226	0.278	0.284	0.280	0.344	0.272	0.235
Xinjiang	0.186	0.153	0.286	0.398	0.363	0.356	0.366	0.365	0.279
Liaoning	0.704	0.665	0.640	0.663	0.687	0.704	0.722	0.738	0.675
Jilin	0.674	0.678	0.749	0.890	0.962	0.930	0.934	0.966	0.784
Heilongjiang	0.800	0.709	0.630	0.663	0.665	0.670	0.681	0.694	0.678

Authors' elaboration.

Firstly, the GTFP in the pharmaceutical industry is markedly different among provinces and regions. For the average GTFP, Table 2 shows that 13.33% of provinces are below 0.6, 60% stay at the 0.6–0.8 level, and 26.67% of provinces have an average GTFP higher than 0.8. The mean GTFP in Jiangsu is the highest (0.892), Tianjin, Beijing, Chongqing, Zhejiang, Shanghai, Guangdong, and Fujian are all higher than 0.8. These provinces have achieved great economic development, and the majority of them have moved into the post-industrialization phase of their own economies. The development of the pharmaceutical sector was helped along by the advantages of urbanization and improved infrastructure. The expansion of the economy has led to advances in technology, and the implementation of environmentally friendly production methods has greatly cut down on pollution. Furthermore, the number of pharmaceutical enterprises in these provinces is more than in others, and the economies of scale also improved the GTFP.

**Figure 2:**
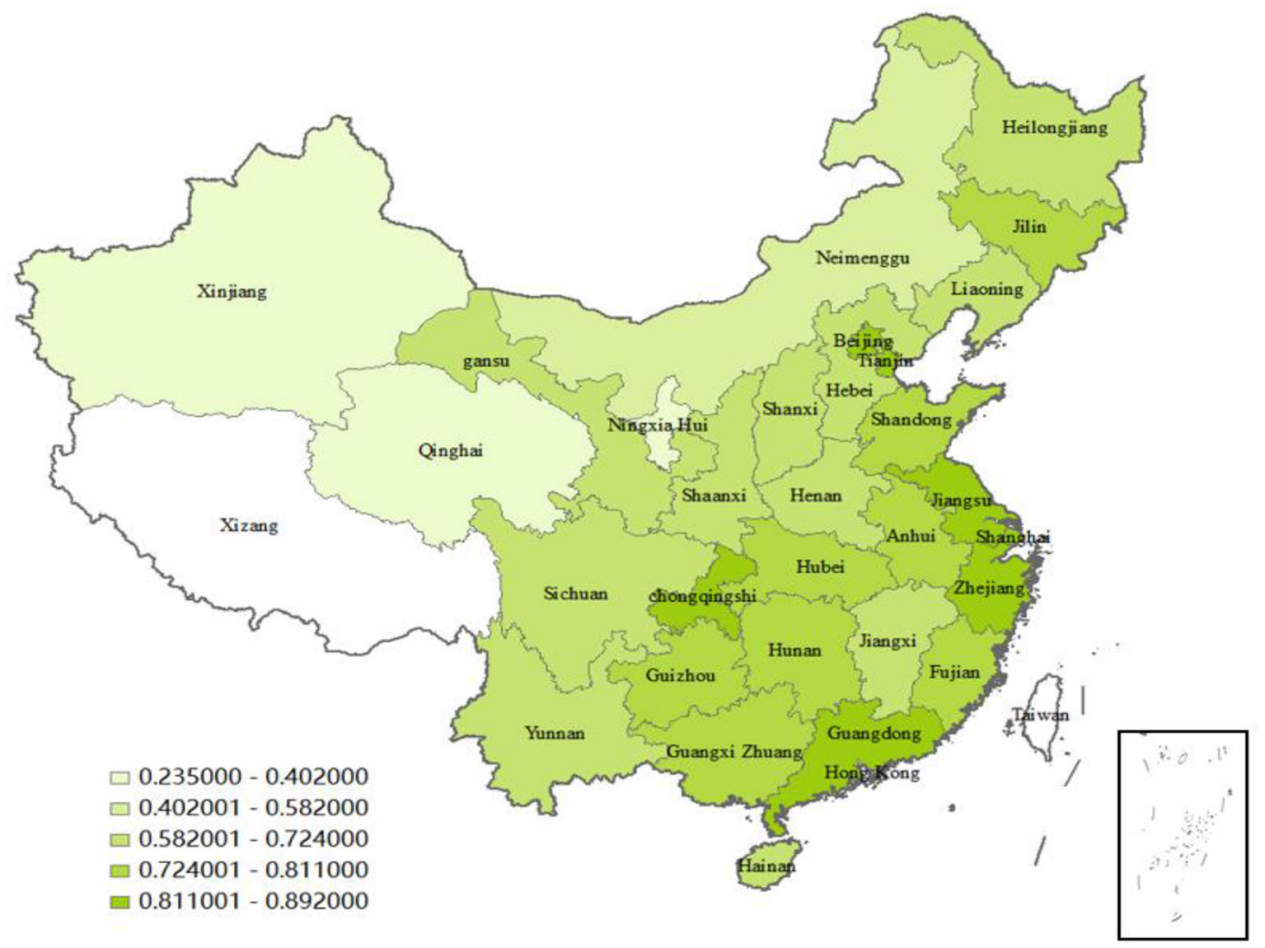
Geographical distributions of the pharmaceutical industry's GTFP in 30 provinces.

The GTFP of Jilin Province is also at a higher level, which is 0.784. Changbai Mountain region in Jilin Province is an important medicinal material-producing area in north China. With a strong medical industry foundation and a high degree of specialization, relying on the rich medicinal material resources in Changbai Mountain, Jilin has vigorously developed the modern pharmaceutical industry and become an important pharmaceutical industry base in northeast China and even in the whole country. Ningxia has the lowest GTFP (0.235), Xinjiang, Qinghai, and Inner Mongolia are both <0.6. Due to the relatively backward economic development of these provinces, most of them are located in mountainous areas with complex terrain and high transportation costs. At the same time, factors such as large areas, sparse population, and high labor costs hinder the development of the pharmaceutical industry in these provinces. There are huge differences among the 30 provinces. Jiangsu, the province with the greatest level, has a mean GTFP that is 3.796 times higher than that of Ningxia, the province with the lowest level. To make the differences among provinces clearly, Figure 2 is a quintile map drawn by ARCGIS, which represents the efficiency of the pharmaceutical industry in different provinces according to color depth.

**Figure 3:**
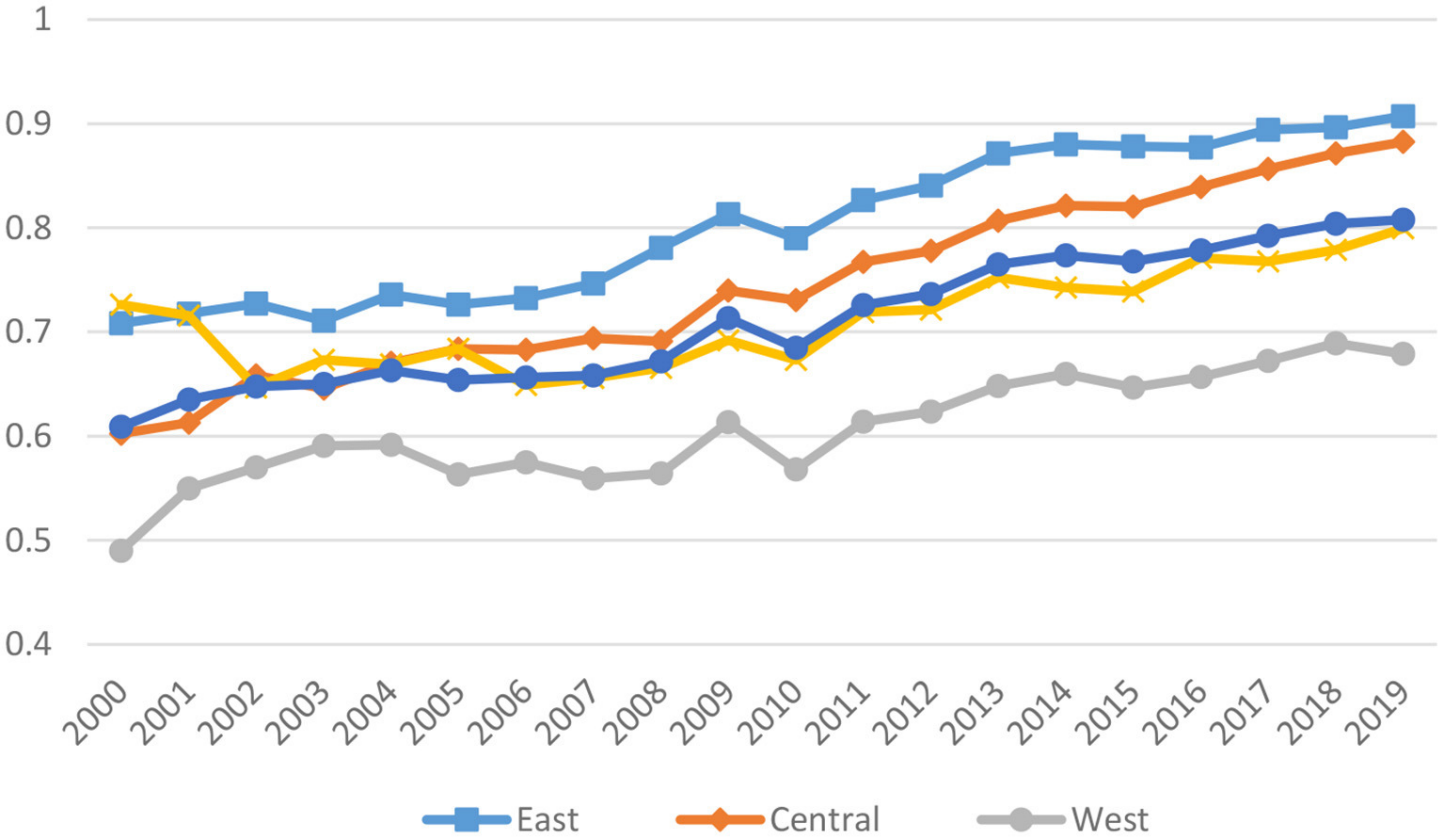
Trends in the GTFP of the pharmaceutical industry in four regions.

Secondly, to further analyze the differences of pharmaceutical industry's GTFP in China's four major regions clearly, this paper calculated the GTFP in each region. As shown in Figure 3, among the four regions, East > Central > Northeast > west. The eastern region occupies an excellent geographical location, is the most economically developed region in China, and the most open region in China, leading other regions in terms of industrial equipment and industrial enterprise management. The tremendous economic strength of the eastern region makes it possible for the region to adopt some beneficial measures to adjust the interaction between energy and the environment, which in turn has a positive influence on the GTFP of the pharmaceutical business. The eastern region has the largest number of pharmaceutical companies, followed by the central region. The central region is located in the hinterland of China, where many institutions of higher learning and relatively dense talents exist. In recent years, the central region has vigorously developed characteristic pharmaceutical professional production bases, and the total pharmaceutical production is second only to the eastern region. Resource industries and capital industries dominate the northeastern region, and the GTFP is lower than that of the eastern and central regions (60). The vast majority of western provinces, on the other hand, are regarded as being economically backward and have unsteady foundations in terms of their business sectors. At this time, there are not nearly enough facilities in the pharmaceutical business that are capable of preserving energy, cutting emissions, and controlling pollutants. As a result, the western region tends to waste more energy and reduce GTFP.

Although the GTFP in most provinces has been improved gradually in the context of a rapid economic boost, the general level is not as high as expected. Energy conservation and emission reduction technologies are relatively inefficient, often resulting in more energy waste and pollution.

### Dynamic analysis of GTFP in China's pharmaceutical industry

The GML index, together with the results of its GEC and GTC decomposition, were calculated from the years 2000 to 2019, and the findings are presented in Table 3. This was done in order to conduct an analysis of the dynamic GTFP in China's pharmaceutical industry.

**Table 3:** Decomposition results of the GML index in China's pharmaceutical industry.

**Year**	**GML**	**GEC**	**GTC**
2000–2001	0.9791	0.9850	0.9940
2001–2002	1.0251	1.0160	1.0090
2002–2003	1.0040	1.0050	0.9990
2003–2004	0.9880	0.9940	0.9940
2004–2005	0.9811	0.9930	0.9880
2005–2006	0.9960	0.9990	0.9970
2006–2007	0.9930	0.9950	0.9980
2007–2008	0.9821	0.9880	0.9940
2008–2009	0.9860	0.9950	0.9910
2009–2010	1.0080	1.0040	1.0040
2010–2011	1.0281	1.0060	1.0220
2011–2012	0.9970	1.0030	0.9940
2012–2013	1.0040	0.9980	1.0060
2013–2014	1.0010	1.0030	0.9980
2014–2015	1.0050	0.9990	1.0060
2015–2016	1.0649	1.0410	1.0230
2016–2017	0.9740	0.9740	1.0000
2017–2018	1.0120	1.0080	1.0040
2018–2019	1.0606	1.0460	1.0140
Mean	1.0047	1.0027	1.0018

First, the GML index in China's pharmaceutical industry showed an overall growth trend from 2000 to 2019, and the changes during the study period had obvious stage characteristics. Before 2010, the fluctuation range was relatively small, with the GML index mostly <1 and the fluctuation range of 0.9791–1.0251. Specifically, from 2000 to 2003, the GML index is over 1, in a rising state. This is because, with the implementation and promotion of reform and opening up, foreign capital entered China's pharmaceutical industry, many joint ventures emerged, and domestic enterprises grew rapidly. At the same time, the government launched a number of policies and measures to rectify the pharmaceutical market and promote the rapid development of the pharmaceutical industry. From 2003 to 2009, the average annual GML index is <1, in a declining state. This is because, after China acceded to the WTO in 2002, the pharmaceutical industry has set off a wave of reorganization to meet the challenges brought by internationalization. Some investments have withdrawn for various reasons, while others have poured into the pharmaceutical industry, leading to a slowdown in the development of the pharmaceutical industry. After 2010, except that the GML index is <1 in 2011–2012 and 2016–2017, the GML index in other years is mostly over 1, and the fluctuation range of GML in 2010–2019 is between 0.9623 and 1.0649. The years with the largest increase were 2015–2016, while the years with the largest decline were 2016–2017. The average national GML index during the sample period is 1.0047, indicating that the average growth rate of the development level of the industry in China is 0.47%. It can be seen that China's pharmaceutical industry has stepped into a mature stage of development after 2010, and the development speed and quality have been significantly improved.

**Figure 4:**
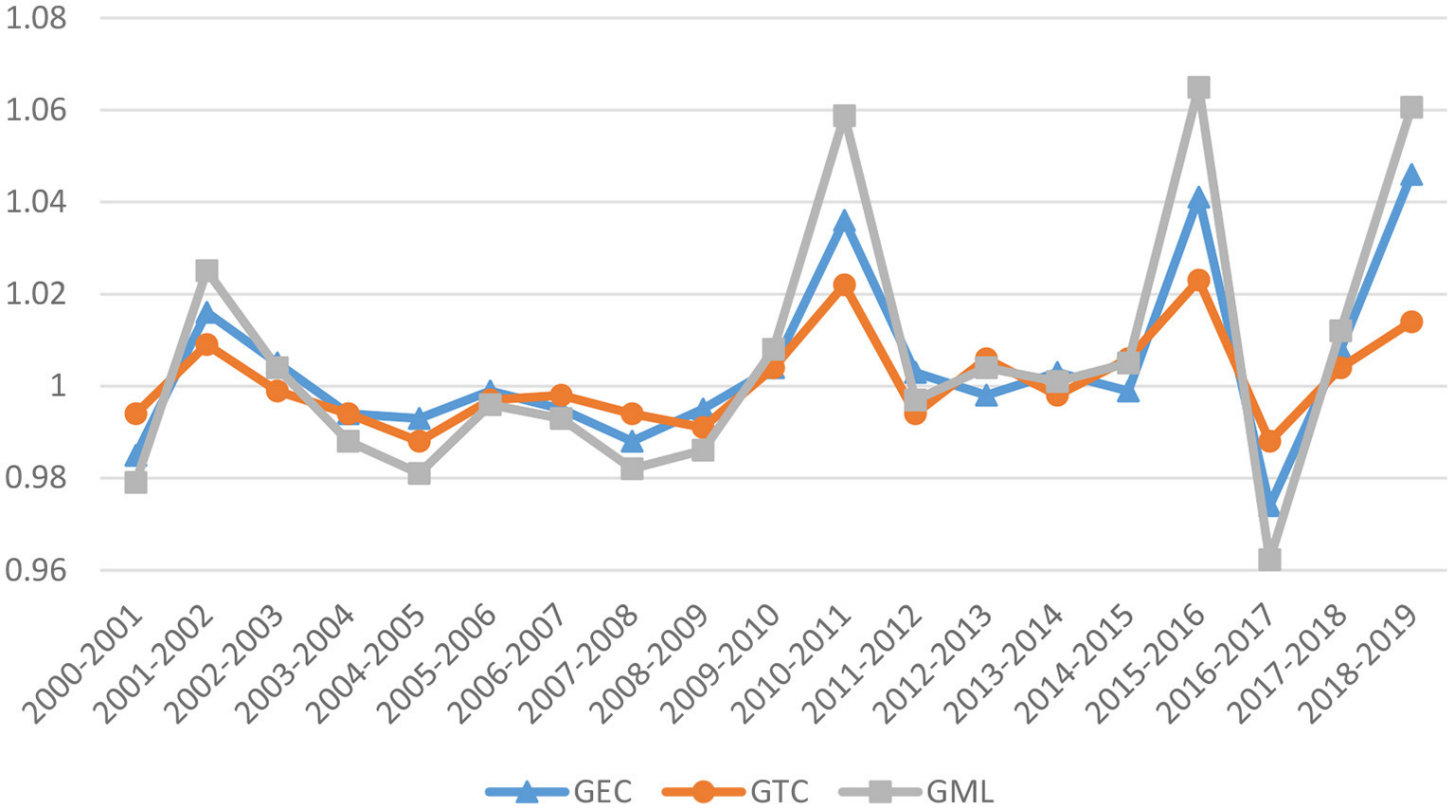
Trends in the GML index and its decomposition in China's pharmaceutical industry.

Further, analyzing the decomposition index GEC and GTC of GML in China's pharmaceutical industry, Figure 4 depicts the average values of these indicators for all of the provinces over the course of the previous few years. Both the GTC and the GML index are exhibiting trends that are virtually comparable from the point of view of the decomposition of the GML index. According to the calculation, the fluctuation range of GEC is relatively small during the sampling period, with an average of 1.0027, and that of GTC is 1.0018. The mean values of these two indicators are both over 1, indicating that they are both in an increasing state. Technological progress and improvement of technical efficiency both promote the development of GML.

In the final part of our research project, we investigate regional variations in how the GML index of China's pharmaceutical business has changed. The GML index as well as the changing mean values of GEC and GTC in China's pharmaceutical business in the four regions are presented in Table 4, which covers the years 2000 through 2019. According to the calculation results, the growth rate of the central region is the highest, which is 0.67%, and China's pharmaceutical industry has shown a trend of shifting from the eastern region to the central region. The increase in the proportion of the pharmaceutical industry and the number of enterprises in the central region indicates that the region is actively undertaking this industry, so the GML index of the pharmaceutical industry in this region has the largest growth. The eastern region's mean value GML index is 1.0060, only lower than the central region. As mentioned above, the eastern region has a good foundation for social and economic development, which provides strong material support for the rapid development of China's pharmaceutical industry. The development of the pharmaceutical industry in the eastern region follows the direction of advanced international technology and high-tech products and constantly carries out industrial upgrading.

**Table 4:** The regional differences in the GML index of China's pharmaceutical industry.

**Region**	**GML**	**GEC**	**GTC**
Eastern region mean	1.0060	1.0023	1.0038
Central region mean	1.0067	1.000	1.0065
Western region mean	0.9940	1.0001	0.9939
Northeastern region mean	0.9980	1.0003	0.9977
National average	1.0047	1.0027	1.0018

The mean values of GML and GEC of the pharmaceutical industry in northeast China are 0.998 and 1.0003, higher than the national average, and GTC is 0.9977, indicating that GTC restricted the development of the pharmaceutical industry in the northeastern. The GML index of the pharmaceutical industry in western is only 0.9940. In contrast, the western region is rich in traditional Chinese medicine resources, but due to the low proportion of the pharmaceutical industry, scattered distribution of large enterprises, and the lagging behind in technological innovation. These reasons have led to the shift of the pharmaceutical industry from the western region, and the current development momentum of the industry is gradually declining.

### Tobit regression results and analysis

#### Tobit regression results

In this paper, the regression results obtained by using the software Stata15.0 are shown in Table 5. Columns (1–3) of Table 5 reflect the results of models (6–8). Column (1) shows that the *er* coefficient is statistically significant and negative with the addition of control variables. The significance is at a 1% level. And the *er2* is significantly significant and positive. It means a U-shaped relationship exists between the environmental regulations and the pharmaceutical industry's GTFP. In China's pharmaceutical industry, when the intensity of environmental regulations remains within a certain “critical point,” it will suppress the GTFP. But when the intensity of environmental regulations exceeds the “critical point,” its impact on GTFP is restrained, and its influence is enhanced. With the intensification of environmental regulations, ecological technology innovation research and development costs less than pollution control costs. In pursuit of profit maximization, pharmaceutical companies often decide to carry out ecological technology innovation after comparing the costs and benefits of various schemes. The research results confirm that implementing compliant environmental regulations will encourage enterprises to improve GTFP and their comprehensive competitiveness.

**Table 5:** Regression results of the Tobit model.

**Variables**	**GTFP**		
	**(1)**	**(2)**	**(3)**
*er*	−0.184***	−0.199***	−0.180***
	(0.0246)	(0.0245)	(0.0264)
*er2*	0.0821***	0.0858***	0.0677***
	(0.0144)	(0.0142)	(0.0169)
*Grd*		0.0130***	0.0113***
		(0.00304)	(0.00315)
er*grd			0.0013*
			(0.0007)
z1	0.0266***	0.0181***	0.0187***
	(0.00518)	(0.00547)	(0.00546)
z2	0.00521***	0.00393***	0.00343***
	(0.00106)	(0.00109)	(0.00111)
z3	0.136***	0.111***	0.109***
	(0.0370)	(0.0369)	(0.0368)
z4	0.0249	0.0169	0.0172
	(0.0172)	(0.0171)	(0.0170)
Constant	0.512***	0.515***	0.523***
	(0.0391)	(0.0385)	(0.0386)

*** and * indicate significance levels at 1 and 10%, respectively, the corresponding *t*-values are in parentheses. Authors' elaboration.

Further, Column (2) shows the results of model (7). There is also a U-shaped relationship between environmental regulations and the pharmaceutical industry's GTFP. The government R&D subsidies to GTFP are positive and significant at 1%, which indicates that after controlling other factors. This means that government R&D subsidies have a positive direct impact on GTFP under environmental regulation policies. The findings are consistent with those found in Howell's research (61). The costs of environmentally friendly innovation are generally greater than the price of general innovation, particularly for the pharmaceutical business. Enterprises can receive financial help from the government in the form of R&D subsidies, which can then be used to engage in R&D activities, thereby lowering costs and stimulating a tendency toward green innovation.

Among them, er^*^grd is the interactive item between environmental regulations and government R&D subsidies, which is used to measure the impact of the simultaneous implementation of the explanatory variables on the GTFP in China's pharmaceutical industry. The results are shown in Columns (3) of Table 5; the coefficient of er^*^grd is significantly positive at the level of 10%, which indicates that the joint effect of environmental regulations and government R&D subsidies is conducive to the improvement of the GTFP in China's pharmaceutical industry (53).

Then, analyzing the regression results of control variables, Openness to the outside world at the significance level of 1% indicates that the higher degree of opening contributes to the improvement of GTFP in China's pharmaceutical industry. This is because openness will lead to higher economic development, scientific research, and technological progress in a region. The urbanization coefficient is 0.00521, and the significance is at a 1% level. Provinces with a high level of urbanization also have better infrastructure, it has a positive impact on GTFP. Then, the GDP direct coefficient is 0.136, at the significance level of 5%. indicating that the current government intervention positively impacts the GTFP. The fact that the GDP has a positive effect on the entire country is evidence that the expansion of the economy as a whole has contributed to an increase in the national income. This means that more funding can be allocated to environmental governance, which is beneficial to the improvement of the environment. At last, the regression coefficient of enterprise-scale is positive but not significant.

#### Robustness check

The main conclusion is drawn by discussing the relationship among environmental regulations, government R&D subsidies, and the pharmaceutical industry's GTFP. To verify the accuracy of the conclusion, it is necessary to check the robustness of the conclusion, and in this paper, we replace the explained variables to verify the robustness of the conclusion.

Following the past practice of most scholars, in this paper, the GTFP is replaced by the TFP (62). We select the input variables, including labor, intermediate, and capital inputs. The number of employees expresses the labor input variable. The intermediate input is expressed with the operating cost. The capital input variable is expressed with a net fixed asset (63). The output variables include the main business income and total profits of 30 provinces in China's pharmaceutical industry.

Table 6 presents the detailed results; after replacing the explained variables, the interaction of environmental regulations and government R&D subsidies on GTFP is consistent with the previous results. Overall, the evaluation results of this research are reliable and robust.

**Table 6:** Regression results.

	**TFP**		
	**(1)**	**(2)**	**(3)**
*Er*	−0.143***	−0.163***	−0.167***
	(0.0435)	(0.0451)	(0.0454)
*er2*	0.0528*	0.0613**	0.0603**
	(0.0275)	(0.0278)	(0.0278)
*Grd*		0.00799	0.00651
		(0.00509)	(0.00547)
er*grd			0.0019*
			(−0.0003)
z1	0.0343***	0.0281***	0.0294***
	(0.00895)	(0.00975)	(0.00992)
z2	0.00693***	0.00609***	0.00572***
	(0.00165)	(0.00173)	(0.00180)
z3	0.207***	0.192**	0.186**
	(0.0790)	(0.0790)	(0.0793)
z4	0.00928	0.00435	−0.000492
	(0.0300)	(0.0300)	(0.0307)
Constant	0.434***	0.448***	0.451***
	(0.0648)	(0.0650)	(0.0650)

***, ** and * indicate significance levels at 1, 5 and 10%, respectively, the corresponding *t*-values are in parentheses. Authors' elaboration.

## Conclusions

The research studies the influence of environmental rules and government R&D subsidies on the GTFP and confirms the degree and direction of the impact based on panel data of 30 provinces in China's pharmaceutical sector from 2000 to 2019. Firstly, static analysis shows that the GTFP in China's pharmaceutical industry is markedly different among provinces and regions. The mean GTFP in Jiangsu is the highest (0.892), and Ningxia has the lowest GTFP of 0.235. 13.33% of provinces GTFP are <0.6, 60% stay at the 0.6–0.8 level of GTFP, and 26.67% of provinces have GTFP higher than 0.8. The eastern zone is the largest and the western region is the smallest, according to the GTFP. The pharmaceutical sector in China exhibits a varying upward tendency in terms of GML's temporal evolution features. Since 2010, the pharmaceutical industry's development speed and quality have significantly improved. This paper also studies the regional differences in the GML index of China's pharmaceutical industry. The central region had the highest growth rate at 0.67%, showing a development trend from east to central. Although China's green economy efficiency and environmental regulation level exhibit obvious spatial variances under the general trend of green development and the collective action of the entire society, the GTFP in each region has exhibited a consistent rising trend over time.

Then, the Tobit model concludes that environmental regulations have a U-shaped relationship with the GTFP in the pharmaceutical industry. The government R&D subsidies to GTFP are positive and significant, and with the expansion of government R&D subsidies, the promotion effect of environmental regulations on GTFP is enhanced. Based on the above conclusions, it can be seen that to promote the GTFP in Chinese pharmaceutical industrial enterprises, environmental regulations and government R&D subsidies are necessary, and the two policies should complement each other.

## Data availability statement

Publicly available datasets were analyzed in this study. This data can be found here: https://data.worldbank.org/.

## Author contributions

The author confirms being the sole contributor of this work and has approved it for publication.

## Conflict of interest

The author declares that the research was conducted in the absence of any commercial or financial relationships that could be construed as a potential conflict of interest.

## Publisher's note

All claims expressed in this article are solely those of the authors and do not necessarily represent those of their affiliated organizations, or those of the publisher, the editors and the reviewers. Any product that may be evaluated in this article, or claim that may be made by its manufacturer, is not guaranteed or endorsed by the publisher.

## Footnotes

^1^Ministry of industry and information technology (MIIT) guideline for pharmaceutical industry development plan (2016).

^2^NBS (National Bureau of Statistics). China statistical yearbook. Beijing: China Statistics Press; 2020.
